# Current Therapeutic Approaches in Metastatic and Recurrent Ewing Sarcoma

**DOI:** 10.1155/2011/863210

**Published:** 2010-12-01

**Authors:** Michael Huang, Kenneth Lucas

**Affiliations:** Division of Pediatric Hematology/Oncology, Department of Pediatrics, H085, Penn State Milton S. Hershey Medical Center, Penn State College of Medicine, Penn State Hershey Children's Hospital, 500 University Drive, P.O. Box 850, Hershey, PA 17033-0850, USA

## Abstract

Ewing sarcoma (ES) is the second most common type of primary bone malignancy in children and young adults. Survival rates for localized ES have improved to upwards of 70% with aggressive chemotherapy and local control. On the other hand, there has been little improvement in survival rates for patients with metastatic or recurrent ES. Herein we review the different current therapeutic approaches available, including the different upfront and salvage chemotherapy regimens, the role for stem cell transplantation, and potential use of immunotherapy.

## 1. Background

Ewing sarcoma (ES) is the second most common type of primary bone malignancy in children and young adults, and age of onset is most often in the second decade, with a slight male predominance [1]. The ES family of tumors is a group of small round blue cell neoplasms of neuroectodermal origin, which includes classical ES, primitive neuroectodermal tumors (PNETs), and Askin tumors of the chest wall. In the pre-chemotherapy era, less than 10% of patients with ES survived. In the current era of multimodality therapy, event free survival (EFS) rates have increased to greater than 70% for localized disease [2, 3]. Conventional treatment regimens for localized ES vary but in general consist of a combination of the following chemotherapeutic agents: vincristine, actinomycin-D, cyclophosphamide, doxorubicin, ifosfamide, and etoposide. Adjunctive surgical resection with or without radiation therapy is used for local control. In North America, the 5-drug regimen of vincristine, doxorubicin, and cyclophosphamide (VDC) alternating with ifosfamide and etoposide (IE) is considered standard. The Children's Oncology Group (COG) has reported a 73% EFS rate utilizing an interval compression strategy with this 5-drug alternating regimen [3]. 

On the other hand, there has been little improvement in survival rates for patients with metastatic or recurrent ES despite aggressive treatment. Approximately 25% of patients present with metastatic disease at diagnosis, with the lung and bone being the most common sites of disease spread. For metastatic ES, the 5-year EFS is approximately 30%, although isolated pulmonary metastasis is associated with better prognosis [4]. 

Most episodes of disease recurrence occur after completion of therapy and most recurrences (approximately 80%) occur within 2 years of initial diagnosis [5]. The time to disease recurrence is the most important indicator of overall survival (OS). Late recurrence (>2 years from diagnosis) carries an OS rate of greater than 25% while early recurrence is associated with an OS rate of less than 10% [6]. Although there has been limited success with current conventional treatment options for both metastatic and recurrent ES, newer therapeutic agents are on the horizon. In this paper, we will review the current therapeutic approaches for both metastatic and recurrent ES, including the different upfront and salvage chemotherapy regimens, the role for stem cell transplantation (SCT), and potential future use of immunotherapy.

## 2. Metastatic Ewing Sarcoma

### 2.1. Systemic Therapy

First-line therapy for metastatic ES is similar to that for localized disease and utilizes the same chemotherapy backbone with adequate local control to both primary and metastatic sites. While this strategy often results in complete or partial responses, OS rates remain dismal at 20% [7]. Attempts to improve outcomes through changes in chemotherapy regimens have been largely unsuccessful. The INT-0091 study from the COG reported no benefit with the addition of IE to a standard backbone of vincristine, actinomycin-D, cyclophosphamide, and doxorubicin (VACD) [8]. In the second Intergroup Ewing Sarcoma Study (IESS-2), the addition of 5-fluorouracil (5-FU) failed to improve outcome in this subset of patients [9]. In a phase II trial from the Pediatric Oncology Group, high-dose alkylator therapy with topotecan or topotecan plus cyclophosphamide did not improve patient outcomes; however, the latter combination did show activity against metastatic disease (response rate of 57%). The combination of topotecan and cyclophosphamide will be further evaluated in future COG trials [10, 11].

### 2.2. Local Control

Currently, upfront whole-lung irradiation is often used in patients with lung metastases, regardless of radiographic response following neoadjuvant chemotherapy. The strongest evidence for this comes from the European Intergroup Cooperative Ewing Sarcoma Study (EICESS) group, which reported an EFS rate of 38% (versus 27% in nonirradiated patients) using 15 to 18 Gy whole lung irradiation in patients with isolated lung metastases [12]. Unlike osteosarcoma, there is little role for pulmonary metastasectomy in these patients. 

Data from the recently concluded EURO-E.W.I.N.G. 99 trial emphasizes the value for aggressive local control for extrapulmonary metastatic ES. Significant improvement in EFS rates was observed with combined surgery and radiation (56% EFS) compared to either modality alone (34% EFS) [13]. Metastatic sites of disease in bone and soft tissues should receive fractionated radiation therapy with total doses of 45 to 56 Gy, although care should be taken in limiting the amount of bone marrow included in the radiation field so as to avoid compromising total doses of systemic chemotherapy [14].

### 2.3. Role of Stem Cell Transplantation (SCT)

The role for high-dose myeloablative chemotherapy with autologous stem cell rescue in patients with metastatic ES at initial diagnosis remains controversial. The rationale for autologous SCT is that ES is highly sensitive to high-dose alkylator therapy, but because of steep dose-response curves, relatively small dose reductions can result in sharp decreases in log tumor cell kill [15]. Some reports have shown improved outcomes with this approach whereas others did not. However, due to the paucity of prospective, randomized trials, studies that do suggest a benefit with this modality are subject to selection bias as only a highly selective subgroup of patients who achieve disease remission are eligible to undergo SCT.

The largest reported experience that suggests a benefit with high dose therapy (HDT) with SCT is from the European Bone Marrow Transplant Registry (EBMTR) data. Although the EFS rate for extrapulmonary metastatic ES was only 21%, improved outcome was observed in the subgroup of patients treated with a busulfan-containing regimen. In those patients that received busulfan in combination with melphalan, 5-year OS rate was 44%, compared with only 23% for patients who did not receive this drug [16]. One prospective Children's Cancer Group (CCG) study investigated the efficacy of melphalan, etoposide and 12-Gy total body irradiation (TBI) with autologous SCT in 32 patients with ES metastatic to the bone/bone marrow. This regimen, however, did not improve survival compared with conventional therapy (2-year EFS of 24%) [17]. The EURO-EWING 99 trial is thus far the largest and only randomized study to compare autologous SCT with conventional therapy for patients with metastatic ES. In their recently published data, they reported improved outcome after HDT in those patients who achieved complete remission prior to HDT (3-year EFS rate of 57%) [18]. This reiterates previous reports of pre-SCT remission status as an important prognostic factor in metastatic ES.

### 2.4. Treatment Options under Clinical Evaluation

Alternate approaches have looked into targeting tumor vasculature using low-dose chemotherapy given over an extended period of time (metronomic chemotherapy). The rationale for this approach is that EWS-FLI1 in ES functions as a promoter for vascular endothelial growth factor (VEGF) [14]. COG has recently concluded a pilot study, AEWS02P1, which adds the antiangiogenic agents vinblastine and celecoxib to the standard backbone of vincristine, actinomycin-D, cyclophosphamide, doxorubicin, ifosfamide, and etoposide (VACD/IE). Results from this study are still pending. The mammalian target of rapamycin (mTOR) pathway is implicated in cell proliferation in certain malignancies including ES. It is also postulated that mTOR may serve as an important auxiliary pathway in tumor angiogenesis. Rapamycin (also known as sirolimus), a highly specific mTOR inhibitor, has demonstrated the ability to arrest ES cell proliferation at the G1 phase and also downregulate levels of EWS/FLI-1 proteins [19]. Also, low-dose rapamycin has been shown to inhibit ES tumor growth by inhibiting neovascularization, possibly via COX-2 suppression [20].

Zoledronic acid is a bisphosphonate that has been shown to be effective as adjunctive treatment in primary and metastatic bone tumors. Its mechanism of action in ES is believed to be via induction of apoptosis and inhibition of primary bone tumor growth through a mechanism that involves the upregulation of osteoprotegerin. Preclinical data in murine xenograft models demonstrated that primary tumor growth is inhibited in 66% of cases with zoledronic acid alone and in 88% of cases when combined with paclitaxel [21].

## 3. Recurrent Ewing Sarcoma

### 3.1. Salvage Therapy

For patients with relapsed ES, the outlook is dismal, with a 5-year OS rate of 10%. Patients whose recurrence occurs after 2 years of diagnosis fare better, with an OS rate closer to 25%. Most salvage regimens incorporate previously used active agents along with newer second-line camptothecin-derived agents. Topotecan and irinotecan are topoisomerase I inhibitors that are derived from the naturally occurring compound camptothecin. Camptothecin-containing regimens have demonstrated efficacy for patients with recurrent ES. While topotecan monotherapy has limited activity against ES, topotecan combined with cyclophosphamide produces response in 30%–35% of patients with relapsed ES [22]. The combination of irinotecan and temozolamide has also demonstrated activity in recurrent ES. The group from Memorial Sloan-Kettering reported a 63% response rate in 19 patients treated with this regimen [23]. Others have shown benefit in the use of carboplatin-based regimens for these patients. One such regimen is ICE (ifosfamide, carboplatin, and etoposide) reinduction which showed an overall response rate of 48% in a CCG study of 21 patients with relapsed ES [24]. The combination of docetaxel and gemcitabine has also been reported to achieve objective responses in cases of recurrent ES [25].

### 3.2. Role of Stem Cell Transplantation (SCT)

HDT followed by autologous SCT may be beneficial for a select subset of patients with recurrent ES who respond favorably to salvage therapy. Data to support this approach, however, is lacking as most studies do not confine their investigation to recurrent ES in particular. A group from the University of Washington investigated this approach specifically in patients with recurrent EFS using busulfan, melphalan, and thiotepa as the conditioning regimen and observed improved survival rates in those patients with chemosensitive recurrent disease (5-year EFS and OS rates of 61% and 77%, resp.) [26]. Allogeneic SCT is a potential alternative strategy for recurrent ES but carries with it the risk for added morbidity. The rationale behind the use of allogeneic SCT is based on the potential immunogenicity of major histocompatibility (MHC) molecules and tumor antigens expressed on ES cells to elicit a graft-versus-tumor effect [27]. There is also preliminary clinical data to suggest that donor NK cells may exert antitumor activity following allogeneic SCT and promotes further investigation into the use of killer cell immunoglobulin-like receptor- (KIR-) HLA-mismatched SCT [28]. In one series from the EICESS group of 17 patients with high-risk ES, four subjects received allogeneic SCT, and three of these subjects experienced long-term disease-free survival [29]. Conversely, Burdach et al. reported no advantage in survival outcome with the use of allogeneic SCT and also documented a much higher rate of transplant-related mortality (40% compared to 19% following autologous SCT) [30]. Our institution previously published a case of a 4-year-old female with recurrent metastatic ES who achieved long-term remission following allogeneic SCT [31].

### 3.3. Immunotherapy and Other Treatment Options under Clinical Evaluation

COG is currently evaluating a number of newer chemotherapeutic agents against ES in phase II trials. Other agents under evaluation include vinorelbine, a semisynthetic vinca alkaloid; trabectedin, which causes DNA backbone cleavage and tumor cell apoptosis by superoxide formation; oxaliplatin, a third generation platinum agent; pemetrexed, a novel antifolate compound.

An increased understanding of the events involved in ES tumorigenesis has allowed investigation into therapies against key targets such as EWS/FLI1, IGF-1, CD99, and angiogenesis pathways. The IGF system contributes to cellular proliferation and immortality in a wide variety of cancers, including ES. In ES, the EWS/FLI1 fusion protein requires the IGF1 receptor for malignant transformation of fibroblasts in vitro [32]. Several phase I and II trials have demonstrated isolated cases of antitumor activity of anti-IGF1R antibodies in ES [33, 34]. COG has an open phase II study (ADVL0821) looking at cixutumumab, an anti-IGF1 receptor monoclonal antibody. Bevacizumab, a monoclonal antibody to VEGF that is FDA approved in certain cancers, is currently under investigation and has shown antitumor activity in xenograft models [20]. Although there is no current known ligand for CD99, in vitro studies on ES cell lines have demonstrated that CD99 binding and silencing by specific antibodies induces rapid tumor cell death. CD99 is also highly expressed in human hematopoietic stem cells as well as in several cell types in the pancreas and gonads and is likely the reason why clinical trials using anti-CD99 antibodies have yet to be attempted [35]. Targeted therapy holds promise not only for improving outcomes for patients with ES but also for reducing exposure to conventional chemoradiotherapy and its side effects. 

The known potential of immune cells to recognize and kill tumor cells has led to the emergence of vaccine therapy strategies for refractory solid tumors including ES. Two cancer/testis (CT) antigens have been identified as being highly expressed in ES cells, namely XAGE-1 and LIPI [36]. These CT antigens are differentiation antigens whose expression is restricted to normal gametogenic tissues and certain tumors. One NIH study looked at vaccination with dendritic cells (DCs) pulsed with tumor cell lysates in ES subjects. DCs are the most potent antigen-presenting cells (APCs) of the immune system and have the ability to activate T, B, and NK cells alike. Their approach, however, did not alter disease outcome, although there was one mixed clinical response in a patient with recurrent ES [37]. Cho et al. reported a complete remission of residual tumor in one patient with ES following autologous SCT and pulsed DC therapy [38]. The use of NK cells is also currently under investigation and has shown cytotoxicity against ES cells both in vitro and in vivo [37]. A recent study from the University of Tennessee demonstrated that ES cells in vitro were exquisitely sensitive to activated NK cells [39]. Phase I trials utilizing NK cell therapy are underway.

## 4. Conclusion

Survival rates with metastatic and recurrent ES continue to be dismal with current therapeutic approaches. As such, further improvements in treatment strategies for these patients are urgently needed. Data to support the use of high-dose therapy with stem cell transplantation is controversial at best due to the lack of prospective, randomized trials. More novel approaches will focus on the use of targeted therapeutic agents which offer the advantage of both increasing cure and limiting toxicity from chemoradiotherapy.
